# Trends in Hospitalizations for Ambulatory Care–Sensitive Conditions During the COVID-19 Pandemic

**DOI:** 10.1001/jamanetworkopen.2022.2933

**Published:** 2022-03-17

**Authors:** Nora V. Becker, Monita Karmakar, Renuka Tipirneni, John Z. Ayanian

**Affiliations:** 1 Division of General Medicine, University of Michigan Medical School, Ann Arbor; 2 Institute for Healthcare Policy and Innovation, University of Michigan, Ann Arbor

## Abstract

**Importance:**

The association of the COVID-19 pandemic with the quality of ambulatory care is unknown. Hospitalizations for ambulatory care–sensitive conditions (ACSCs) are a well-studied measure of the quality of ambulatory care; however, they may also be associated with other patient-level and system-level factors.

**Objective:**

To describe trends in hospital admissions for ACSCs in the prepandemic period (March 2019 to February 2020) compared with the pandemic period (March 2020 to February 2021).

**Design, Setting, and Participants:**

This cross-sectional study of adults enrolled in a commercial health maintenance organization in Michigan included 1 240 409 unique adults (13 011 176 person-months) in the prepandemic period and 1 206 361 unique adults (12 759 675 person-months) in the pandemic period.

**Exposure:**

COVID-19 pandemic (March 2020 to February 2021).

**Main Outcomes and Measures:**

Adjusted relative risk (aRR) of ACSC hospitalizations and intensive care unit stays for ACSC hospitalizations and adjusted incidence rate ratio of the length of stay of ACSC hospitalizations in the prepandemic (March 2019 to February 2020) vs pandemic (March 2020 to February 2021) periods, adjusted for patient age, sex, calendar month of admission, and county of residence.

**Results:**

The study population included 1 240 409 unique adults (13 011 176 person-months) in the prepandemic period and 1 206 361 unique adults (12 759 675 person-months) in the pandemic period, in which 51.3% of person-months (n = 6 547 231) were for female patients, with a relatively even age distribution between the ages of 24 and 64 years. The relative risk of having any ACSC hospitalization in the pandemic period compared with the prepandemic period was 0.72 (95% CI, 0.69-0.76; *P* < .001). This decrease in risk was slightly larger in magnitude than the overall reduction in non-ACSC, non–COVID-19 hospitalization rates (aRR, 0.82; 95% CI, 0.81-0.83; *P* < .001). Large reductions were found in the relative risk of respiratory-related ACSC hospitalizations (aRR, 0.54; 95% CI, 0.50-0.58; *P* < .001), with non–statistically significant reductions in diabetes-related ACSCs (aRR, 0.91; 95% CI, 0.83-1.00; *P* = .05) and a statistically significant reduction in all other ACSC hospitalizations (aRR, 0.79; 95% CI, 0.74-0.85; *P* < .001). Among ACSC hospitalizations, no change was found in the percentage that included an intensive care unit stay (aRR, 0.99; 95% CI, 0.94-1.04; *P* = .64), and no change was found in the length of stay (adjusted incidence rate ratio, 1.02; 95% CI, 0.98-1.06; *P* = .33).

**Conclusions and Relevance:**

In this cross-sectional study of adults enrolled in a large commercial health maintenance organization plan, the COVID-19 pandemic was associated with reductions in both non-ACSC and ACSC hospitalizations, with particularly large reductions seen in respiratory-related ACSCs. These reductions were likely due to many patient-level and health system–level factors associated with hospitalization rates. Further research into the causes and long-term outcomes associated with these reductions in ACSC admissions is needed to understand how the pandemic has affected the delivery of ambulatory and hospital care in the US.

## Key Points

**Question:**

How has the rate of potentially preventable hospitalizations (ambulatory care–sensitive conditions) changed during the COVID-19 pandemic?

**Findings:**

This cross-sectional study found statistically significant decreases in rates of potentially preventable hospitalizations during the pandemic relative to the prepandemic period but no significant change in the intensity of care provided during these admissions. These decreases are similar in magnitude to the overall decrease in non–COVID-19–related hospital admissions during the pandemic.

**Meaning:**

This study suggests that researchers should be cautious in interpreting changes in rates of potentially preventable hospitalizations during the pandemic as being solely due to the quality of ambulatory care because these rates are also likely due to other patient-level and hospital-level factors that are associated with the demand for hospital services.

## Teaser

This cross-sectional study describes trends in hospital admissions for ambulatory care–sensitive conditions in the prepandemic period (March 2019 to February 2020) compared with the pandemic period (March 2020 to February 2021).

## Introduction

The COVID-19 pandemic has caused substantial disruptions of routine medical care in the United States, but little is known about the magnitude of these disruptions or their association with longer-term outcomes for patients. During the initial pandemic-related shutdowns in 2020, hospitals and emergency departments saw a sharp decrease in visits and admissions for other non–COVID-19–related care, including for emergencies such as myocardial infarctions and strokes.^1^ Anecdotal reports and articles in the lay press expressed concern that patients were delaying urgent or emergency care out of fear of contracting COVID-19.^2^

Several studies have examined the delays in care and use of non–COVID-19–related medical services during the pandemic. Initial studies reported reductions in hospitalization rates in the spring of 2020.^3^ Two studies that surveyed patients found that 40% to 50% of respondents reported experiencing delays in care during the summer of 2020.^4,5^ Other work examining use of non–COVID-19–related services has found decreases in the use of preventive care services, such as breast and cervical cancer screening, sexually transmitted infection screening, vaccinations, testing for diabetes, and elective surgical procedures.^6,7^

It is not clear whether these delays have translated into decreased quality of care or worse health outcomes for patients. Hospital admissions for ambulatory care–sensitive conditions (ACSCs), defined as admissions for medical problems that are potentially avoidable if they are effectively managed in the outpatient setting, are a common and well-accepted measure of access to and quality of ambulatory care.^8^ Rates of ACSCs have been tracked in numerous countries as a measure of health care quality,^9^ and in 1993, the Institute of Medicine recommended the use of ACSCs to measure the quality of health care over time.^10^ However, the use of ACSCs in drawing conclusions about the quality of ambulatory care has recently been called into question, with a recent study finding that ACSC hospitalization rates were closely associated with other factors, such as patient sociodemographic characteristics, which were in turn associated with overall admission rates.^11^ As a result, some have questioned the common practice of attributing changes in ACSC hospitalization rates to changes in the quality of outpatient care.^12,13^

In this cross-sectional study, we examine the trends in admissions for ACSCs among a commercially insured population in Michigan during the pandemic and compare them with the trends in ACSCs during the prepandemic period. Because some research and news reports have suggested that the pandemic may have caused some patients to delay their presentation to the hospital and therefore require more intensive care once they arrive,^14,15^ we examined both the extensive (overall rates of ACSC hospitalizations) and intensive (length of stay and intensive care unit [ICU] stays among ACSC hospitalizations) margins of ACSC hospitalizations to understand changes in ACSC hospitalizations during the pandemic. To our knowledge, this study is among the first to examine trends in ACSC hospitalizations since the start of the COVID-19 pandemic.

## Methods

### Data and Study Population

In this cross-sectional study, we performed a retrospective analysis of deidentified administrative claims data from the health maintenance organization (HMO) network of Blue Cross Blue Shield of Michigan (BCBSM). Claims for all adults 18 years or older from February 2019 to March 2021 were used to identify hospitalizations. Individuals 65 years or older were included if they or their spouses remained employed and had their primary health coverage through their employer or had purchased a supplementary Medicare plan (Medigap) within the BCBSM HMO network. Medicare Advantage enrollees and traditional Medicare enrollees without Medigap coverage through the BCBSM HMO network were not included. The data were accessed via the Michigan Value Collaborative, a partnership between Michigan hospitals and BCBSM.^16^ Support for the Michigan Value Collaborative is provided by BCBSM as part of the BCBSM Value Partnerships program. This project was approved as secondary use of administrative data with a waiver of informed consent by the University of Michigan institutional review board. The study was reported according to the Strengthening the Reporting of Observational Studies in Epidemiology (STROBE) reporting guideline for cross-sectional studies.^17^

The insurance claims data include comprehensive medical and prescription drug claims for all enrollees, along with enrollee sex, date of birth, and zip code of residence. The raw data files are organized by the month of billing, not the month of service, so all health care use was restructured by service date prior to analysis. Approximately 95% of services delivered in a given month are billed within 3 months. The final month of currently available data is May 2021; therefore, claims that were delivered in February 2021 and billed within 3 months of the service date are included in the study.

### Inclusion and Exclusion Criteria

Our analytic cohort included all BCBSM HMO enrollees aged 18 years or older who were enrolled for any period between March 2019 and February 2021. We excluded individuals with missing or out-of-state zip codes and individuals with missing data on sex or date of birth. Patients 65 years or older were included in our main analysis and excluded in a sensitivity analysis. We also used the zip code of residence to assign enrollees to Michigan counties using a crosswalk from the US Department of Housing and Urban Development.^18^ When zip codes crossed county boundaries, we assigned enrollees to the county where most of the residents within the zip code reside. Details of cohort construction are available in the eFigure in the Supplement.

### Identifying Hospitalizations

Inpatient hospitalizations were identified using a validated algorithm to identify episodes of care.^19^ Each hospitalization was identified using the first date of service, and separate admissions were identified using a washout period of 1 calendar day between the final date of service and the next first date of service for an inpatient claim. Claims for services rendered during the same hospitalization event were deduplicated using a deidentified beneficiary identifier and service dates. For all hospitalizations, we identified the length of stay using the earliest and latest continuous dates of service. We also identified whether the hospitalization included an ICU stay using an algorithm previously validated for administrative claims data.^20^

Hospitalizations were categorized as an ACSC hospitalization; a COVID-19 hospitalization; or a non–COVID-19, non-ACSC hospitalization. Hospitalizations were classified as a COVID-19 hospitalization if the patient’s inpatient claims included a confirmed COVID-19 diagnosis (using *International Statistical Classification of Diseases and Related Health Problems, Tenth Revision* code U071) in any position on the claim. Hospitalizations for ACSCs were identified using an adapted version of the Agency for Healthcare Research and Quality’s available algorithm, which has been validated in Medicare claims.^21^

Ambulatory care–sensitive condition hospitalizations were further classified into 1 of 10 possible ACSC categories, which were then subsequently grouped into 3 categories for our main analyses: diabetes-related ACSC admissions (short-term diabetes complications, long-term diabetes complications, uncontrolled diabetes, and lower extremity amputations related to diabetes), respiratory-related ACSC admissions (asthma in adults aged 18-39 years, asthma or chronic obstructive pulmonary disease [COPD] in adults aged ≥40 years, and community-acquired pneumonia), and other ACSC admissions (hypertension, heart failure, and urinary tract infection). These 3 categories of ACSC hospitalizations were chosen post hoc to reduce the number of hypothesis tests and to increase our power to detect changes over time because the number of ACSC hospitalizations in each of the individual 10 categories was small. However, we also examined and reported trends in each of the 10 individual ACSC hospitalization categories. Further details and sources for all classification algorithms are detailed in eTable 1 in the Supplement.

### Statistical Analysis

#### Descriptive Analyses

For each hospitalization category, we calculated monthly hospitalization rates per 1000 enrollees in the prepandemic period (March 2019 to February 2020) and the pandemic period (March 2020 to February 2021). Hospitalizations were assigned to month by their first date of service, and monthly rates included more than 1 hospitalization per person if the admission dates took place in the same month. For each type of hospitalization, we also calculated the mean and median length of stay by month and the percentage of hospitalizations that included an ICU stay by month. Two ACSC hospitalization categories are age specific (asthma for adults aged 18-39 years and asthma or COPD for adults aged ≥40 years), and the denominators for these were restricted to enrollees in each respective age range.

#### Regression Analyses

General linear regression with a log link and cluster-robust SEs was used to compare the adjusted relative risk (aRR) of an enrollee having a hospitalization in a given month in the prepandemic vs the pandemic period for the following outcomes: any non-ACSC, non–COVID-19 hospitalization; any ACSC-related hospitalization; any respiratory-related ACSC hospitalization; any diabetes-related ACSC hospitalization; and any other ACSC hospitalization. Covariates included patient age, patient sex, and a set of calendar-month and county-level fixed effects, and SEs were robust and clustered at the individual level. These analyses were performed at the person-month level to estimate the aRR that an individual has in a given hospitalization in a given month.

Using a second data set constructed at the admission-month level and restricted to only ACSC hospitalizations, we estimated the aRR that an individual with an ACSC hospitalization would have an ICU stay in the prepandemic vs pandemic periods. We also used negative binomial regression to estimate the adjusted incidence rate ratio of the length of stay of ACSC hospitalizations in the prepandemic vs pandemic periods. These regressions use the same covariates, except for county-level fixed effects, which were excluded because of concerns about power limitations, and clustered robust SEs at the individual level.

To check the robustness of the findings, we conducted several sensitivity analyses. First, we restricted the sample to those aged 18 to 64 years because individuals 65 years or older in our data set may not be representative of the older adult population in the US, which also includes enrollees in Medicare Advantage and those who are dually eligible for Medicare and Medicaid.^22^ Second, we excluded the months of March to May 2020 from our analyses to exclude the first 3 months of the pandemic, when all health care use decreased dramatically. For this analysis, we also excluded March to May 2019 from the prepandemic period to balance the number of months in the prepandemic and pandemic periods. Third, to examine whether our findings were due to compositional changes in the enrolled population during the pandemic, we restricted our sample to only individuals who were continuously enrolled for the entire study period. All analyses were performed using Stata-MP statistical software, version 16 (StataCorp LLC). All *P* values were from 2-sided tests and results were deemed statistically significant at *P* < .05.

## Results

The analytic cohort included 1 240 409 unique adults (13 011 176 person-months) in the prepandemic period (March 2019 to February 2020) and 1 206 361 unique adults (12 759 675 person-months) in the pandemic period (March 2020 to February 2021). In the prepandemic period, 51.2% of enrollees were female, and the age distribution was 12.1% aged 18 to 24 years, 20.1% aged 25 to 34 years, 16.0% aged 35 to 44 years, 17.6% aged 45 to 54 years, 20.2% aged 55 to 64 years, and 14.1% aged 65 years or older; these percentages were very similar in the pandemic period. Table 1 reports the demographic breakdown of person-months by age and sex, as well as the numbers and types of all hospitalizations, COVID-19 hospitalizations, and ACSC hospitalizations. We identified 53 113 person-months with a hospitalization (4.08 per 1000 enrollees) and 55 213 unique hospitalizations in the prepandemic period, and we identified 46 090 person-months with a hospitalization (3.61 per 1000 enrollees) and 47 975 unique hospitalizations in the pandemic period. Individuals could have more than 1 hospitalization in a given month, so the person-month hospitalization rates are slightly lower than the total number of admissions. Among person-months with a hospitalization, 5444 (0.43 per 1000 enrollees) and 3973 (0.31 per 1000) were classified as ACSC hospitalizations in the prepandemic and pandemic periods, respectively. Approximately 40% of ACSC admissions included an ICU stay (4.0% and 3.4% of all admissions in the prepandemic and pandemic periods, respectively). The median length of stay for ACSC hospitalizations was 4 days (IQR, 3-6 days) in the prepandemic period and 4 days (IQR, 3-7 days) in the pandemic period. The eFigure in the Supplement describes the details of the analytic cohort construction, including rates of missing data for all covariates.

**Table 1.** Summary Statistics

Characteristic	Prepandemic period (March 2019 to February 2020)	Pandemic period (March 2020 to February 2021)
Total unique individuals, No.	1 240 409	1 206 361
Total person-months, No.	13 011 176	12 759 675
Age categories in person-months, No. (%)		
18-24 y	1 576 440 (12.1)	1 529 330 (12.0)
25-34 y	2 612 917 (20.1)	2 593 684 (20.3)
35-44 y	2 070 274 (16.0)	2 039 726 (16.0)
45-54 y	2 286 884 (17.6)	2 172 610 (17.0)
55-64 y	2 627 085 (20.2)	2 532 428 (19.9)
≥65 y	1 837 576 (14.1)	1 891 897 (14.8)
Sex in person-months, No. (%)		
Male	6 338 847 (48.7)	6 212 444 (48.7)
Female	6 672 329 (51.2)	6 547 231 (51.3)
Healthcare use in person-months, No. (rate per 1000)^a^		
All hospitalizations	53 113 (42.8)	46 090 (38.2)
COVID-19 hospitalizations	NA	3488 (2.9)
ACSC hospitalizations	5444 (4.4)	3973 (3.3)
Respiratory-related ACSC hospitalizations		
Total	2013 (1.6)	1102 (0.9)
Asthma (ages 18-39 y)	122 (0.1)	63 (0.1)
Asthma or COPD (ages >40 y)	1039 (0.8)	519 (0.4)
Pneumonia	1039 (0.8)	613 (0.5)
Diabetes-related ACSC hospitalizations	991 (0.8)	894 (0.7)
Uncontrolled diabetes	137 (0.1)	124 (0.1)
Diabetes with short-term complications	334 (0.3)	311 (0.3)
Diabetes with long-term complications	486 (0.4)	435 (0.4)
Lower extremity amputations due to diabetes	127 (0.1)	119 (0.1)
Other ACSC hospitalizations	2503 (2.0)	2020 (1.7)
Hypertension	205 (0.2)	172 (0.1)
Heart failure	1810 (1.5)	1487 (1.2)
Urinary tract infection	506 (0.4)	385 (0.3)
ACSC hospitalizations (admissions), No. (%)		
All hospitalizations	55 213 (100)	47 975 (100)
Total ACSC hospitalizations	5557 (10.0)	4056 (8.5)
ICU stay during ACSC hospitalization	2229 (4.0)	1615 (3.4)
LOS for ACSC hospitalizations, median (IQR), d	4 (3-6)	4 (3-7)

Abbreviations: ACSC, ambulatory care–sensitive condition; COPD, chronic obstructive pulmonary disease; ICU, intensive care unit; LOS, length of stay; NA, not applicable.

^a^ Individuals can have more than 1 hospitalization in a given month, so the person-month hospitalization rates are slightly lower than the total number of admissions.

**Figure 1.**
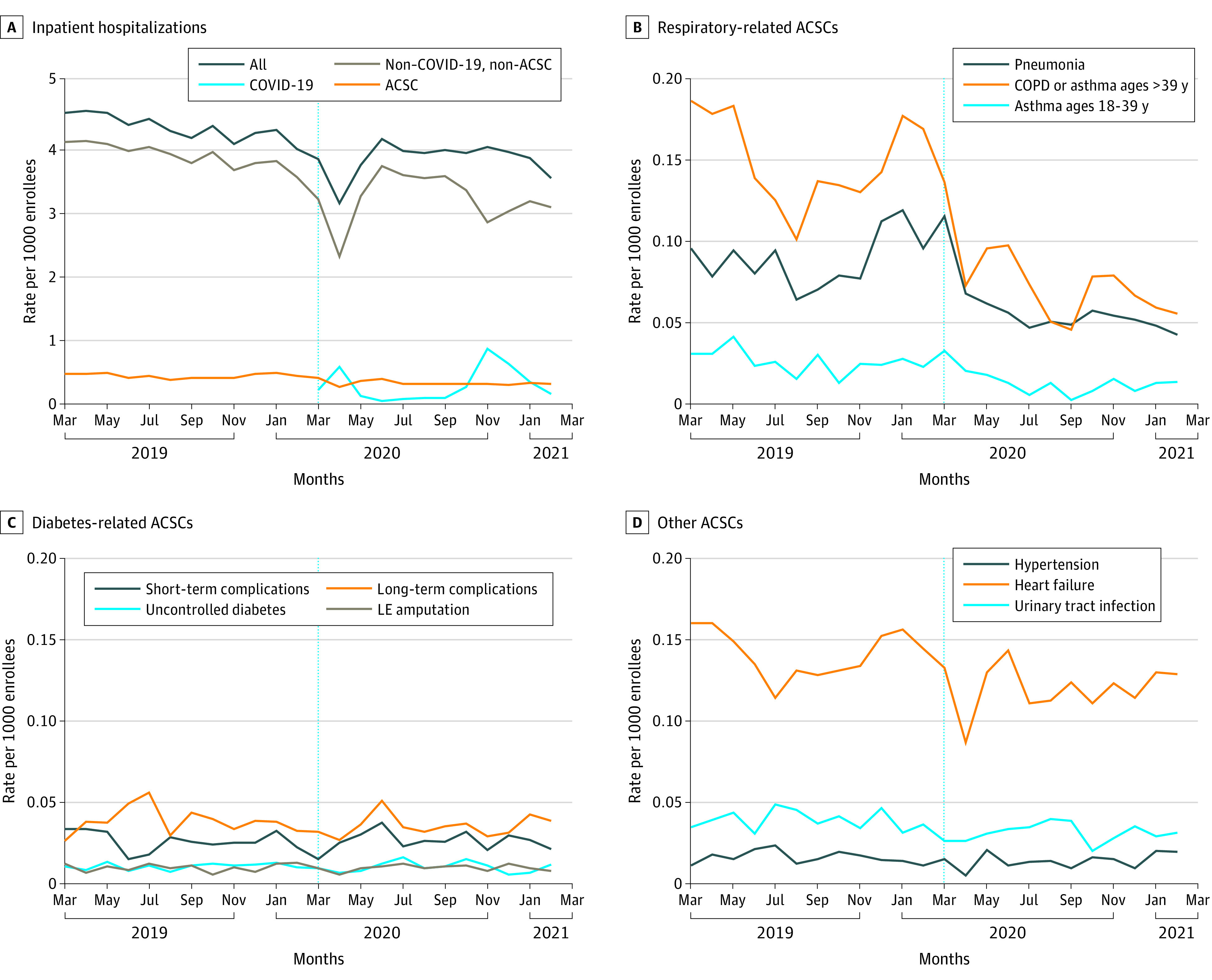
Hospitalization Rates Before and During the COVID-19 Pandemic A, Inpatient hospitalizations. B, Respiratory-related ambulatory care–sensitive conditions (ACSCs). C, Diabetes-related ACSCs. D, Other ACSCs. COPD indicates chronic obstructive pulmonary disease; LE, lower extremity. The vertical dotted line indicates the start of the pandemic in March 2020.

Figure 1 displays monthly hospitalization rates per 1000 enrollees by type of hospitalization. Rates of all hospitalizations ranged from 3.09 to 4.50 hospitalizations per 1000 enrollees in the prepandemic period, decreased to 3.07 hospitalizations per 1000 enrollees in April 2020, and subsequently increased, ranging from 3.46 to 4.07 hospitalizations per 1000 enrollees between June 2020 and February 2021 (Figure 1A). COVID-19 hospitalizations first appeared in the data in March 2020 and ranged from 0.03 to 0.84 hospitalizations per 1000 enrollees in the pandemic period (Figure 1A). Ambulatory care–sensitive condition hospitalizations remained relatively stable overall (range, 0.36-0.47 per 1000 enrollees in the prepandemic period), with a slight decrease in the pandemic period (range, 0.25-0.39 per 1000 enrollees) (Figure 1A).

The hospitalization rates for respiratory-related ACSCs decreased by 30% to 50% in the pandemic period relative to the prepandemic period (Figure 1B). In contrast, changes were not evident for diabetes-related ACSCs (Figure 1C) or other ACSCs (Figure 1D), although there was a decrease in heart failure admissions in April 2020, which appeared to return to the baseline rate (Figure 1D).

**Figure 2.**
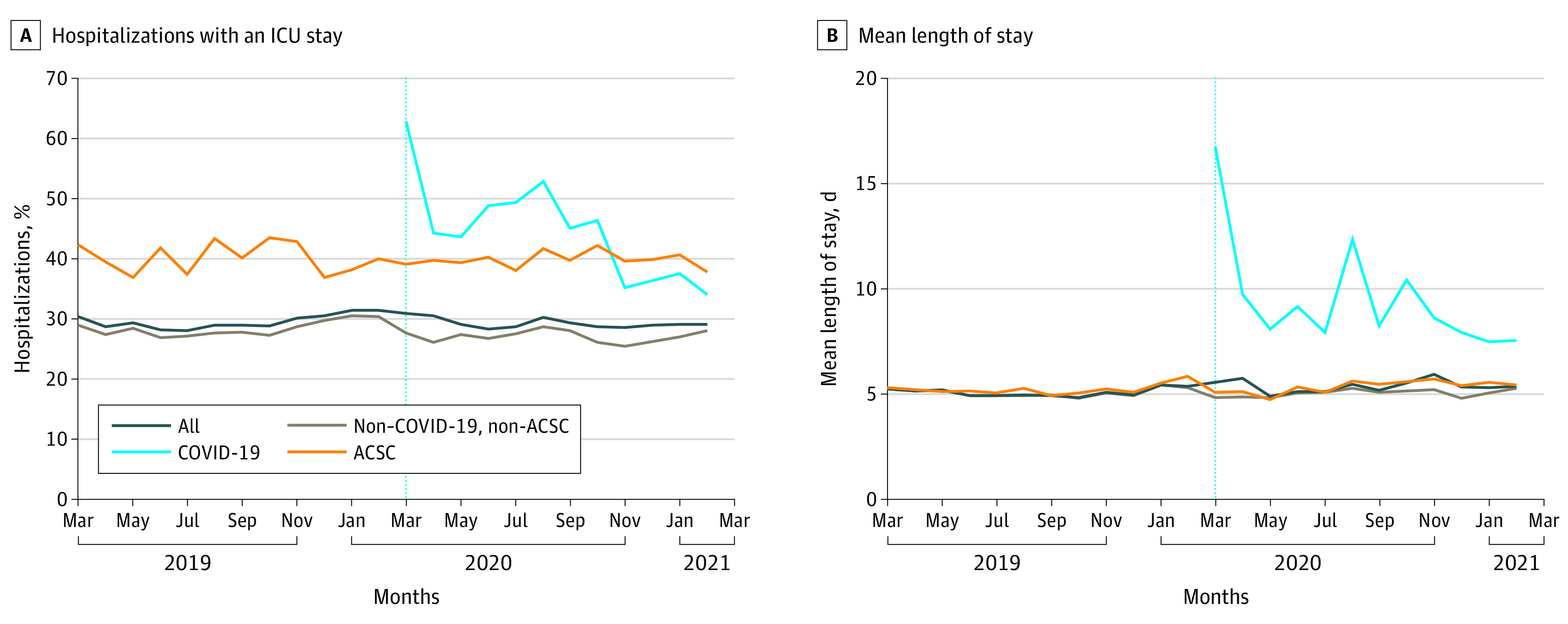
Characteristics of Ambulatory Care–Sensitive Condition (ACSC) Hospitalizations Before and During the Pandemic ICU indicates intensive care unit. The vertical dotted line indicates the start of the pandemic in March 2020.

The percentage of COVID-19 hospitalizations that included an ICU stay decreased during the pandemic period; however, the percentage of ACSC hospitalizations that included an ICU stay remained stable at approximately 40% during the study period (Figure 2A). Similarly, the mean length of stay for COVID-19 hospitalizations decreased during the pandemic period, while the mean length of stay for all other types of hospitalizations remained stable at approximately 5 days (Figure 2B).

Overall, the relative risk of an individual having any non-ACSC, non–COVID-19 hospitalization in the pandemic period compared with the prepandemic period was 0.82 (95% CI, 0.81-0.83; *P* < .001), while the relative risk of ACSC hospitalization was 0.72 (95% CI, 0.69-0.76; *P* < .001) (Table 2). This effect size was associated mostly with reductions in the adjusted odds of respiratory-related ACSC hospitalizations (aRR, 0.54; 95% CI, 0.50-0.58; *P* < .001), with a nonsignificant reduction in diabetes-related ACSCs (aRR, 0.91; 95% CI, 0.83-1.00; *P* = .05) and a statistically significant reduction in other ACSC hospitalizations (aRR, 0.79; 95% CI, 0.74-0.85; *P* < .001). These results did not change substantially in sensitivity analyses when we excluded individuals 65 years or older (eTable 2 in the Supplement) or when we excluded the months of March to May from our analysis (eTable 4 in the Supplement). When we restricted our analysis to the continuously enrolled population, we found statistically significant reductions in all hospitalization categories that were of slightly larger magnitude than those from our baseline models (eTable 6 in the Supplement).

**Table 2.** Adjusted Risk Ratios of ACSC Hospitalizations During the Prepandemic Relative to the Pandemic Period^a^

Characteristic	Any non–COVID-19, non-ACSC hospitalization	ACSC hospitalization			
		Any	Respiratory	Diabetes	Other
No. of person-months	25 770 851	25 759 517	25 602 737	25 539 836	25 675 555
Pandemic relative to prepandemic period, adjusted risk ratio (95% CI)	0.82 (0.81-0.83)	0.72 (0.69-0.76)	0.54 (0.50-0.58)	0.91 (0.83-1.00)	0.79 (0.74-0.85)
*P* value	<.001	<.001	<.001	.05	<.001

Abbreviation: ACSC, ambulatory–care sensitive condition.

^a^ Each model uses data at the person-month level and displays the adjusted risk ratio that an individual enrolled in a given month has in a given hospitalization in 1 of the following categories (non-ACSC, non–COVID-19; ACSC; respiratory-related ACSC; diabetes-related ACSC; or other ACSC) in the prepandemic period (March 2019 to February 2020) compared with the pandemic period (March 2020 to February 2021). Additional covariates include age, sex, and a set of calendar month and Michigan county of residence fixed effects.

Among ACSC hospitalizations, there was no change in the likelihood of an ICU stay (aRR, 0.99; 95% CI, 0.94-1.04; *P* = .64) nor in length of stay (adjusted incidence rate ratio, 1.02; 95% CI, 0.98-1.06; *P* = .33) (Table 3). These results did not significantly change in sensitivity analyses when we excluded individuals 65 years or older (eTable 3 in the Supplement), when we excluded the months of March to May (eTable 5 in the Supplement), or when we restricted analyses to only continuously enrolled individuals (eTable 7 in the Supplement).

**Table 3.** Characteristics of ACSC Hospitalizations Prepandemic Relative to the Pandemic Period^a^

Characteristic	ICU stay for ACSC hospitalizations	LOS for ACSC hospitalization
No. of ACSC admissions	9613	9613
Pandemic relative to prepandemic period	aRR, 0.99 (95% CI 0.94-1.04)	aIRR, 1.02 (95% CI, 0.98-1.06)
*P* value	.64	.33

Abbreviations: ACSC, ambulatory care–sensitive condition; aIRR, adjusted incidence rate ratio; aRR, adjusted risk ratio; ICU, intensive care unit; LOS, length of stay.

^a^ Each model uses data at the ACSC admission level and displays the aRR that an ACSC hospitalization includes an ICU stay and the aIRR of the LOS of that ACSC admission in the prepandemic period (March 2019 to February 2020) compared with the pandemic period (March 2020 to February 2021). Additional covariates include age, sex, and a set of calendar month of admission fixed effects.

## Discussion

We found overall reductions in both non-ACSC and ACSC hospitalizations since the start of the pandemic compared with the prepandemic period among a commercially insured population in Michigan. The reduction in ACSC hospitalizations was associated with large reductions in respiratory-related ACSC hospitalizations, including those for community-acquired pneumonia, asthma, and COPD, but we also found smaller reductions in the odds of other ACSC hospitalizations. To our knowledge, our study is among the first to examine changes in ACSC hospitalizations during the COVID-19 pandemic.

We found no change in the length of stay or in the rates of ICU admissions among ACSC hospitalizations, which suggests that patients who were hospitalized for ACSCs during the pandemic did not appear to require more intensive medical care once they were hospitalized. This is in contrast to research examining other clinical conditions requiring hospitalization, such as stroke, which found that some patients were presenting in more advanced stages of disease and therefore required more intensive care after their arrival.^14^

Because the decreases that we observed in ACSC hospitalization rates were only somewhat larger than the overall decreases seen in non-ACSC hospitalizations, we are cautious about interpreting the reductions that we found in ACSC hospitalizations as solely the result of the quality of ambulatory care. Rather, it seems likely that the pandemic also created many changes in patient behavior and access to care that could have been associated with rates of ACSC hospitalizations, even in the absence of a change in the quality of ambulatory care delivery.

The reduction in respiratory-related ACSC hospitalizations was likely because of a combination of factors. Many individuals with asthma or COPD likely stayed home and complied with masking and social distancing recommendations to avoid contracting COVID-19, resulting in potentially less overall exposure to viral and allergic triggers. There are also some preliminary data that adherence to asthma medications improved at the start of the pandemic, perhaps because patients with chronic respiratory disease were particularly concerned about their risk of COVID-19 infection and correspondingly improved their own asthma management as a result.^23^ Finally, it is also possible that outpatient physicians changed their management of asthma and COPD during the pandemic, working harder to prevent the admission of individuals with high-risk respiratory conditions to reduce their exposure risk. Our finding is consistent with the pediatric literature, which has found that childhood asthma outcomes have improved during the pandemic, including a reduction in pediatric asthma hospitalizations.^24^ Other studies in the United Kingdom have also reported reduced rates of asthma exacerbations in the outpatient and inpatient settings.^25^

Our results may also be partly explained by classification changes. If individuals with chronic respiratory illness were infected with COVID-19 and subsequently hospitalized, their principal diagnosis was likely to be COVID-19, even if their COVID-19 infection was accompanied by an exacerbation of their chronic respiratory disease, and their hospitalization would thus no longer be classified as an ACSC hospitalization.

The smaller reductions that we observed in diabetes-related hospitalizations may also reflect the fact that prepandemic rates for diabetes-related ACSC hospitalizations were much lower than for some other ACSC conditions. If patients and clinicians were already effectively preventing admissions for diabetes before the pandemic, there would be a “floor effect” in the data (ie, there would be fewer opportunities to change care or improve care to lower the diabetes-related ACSC hospitalization rates further once the pandemic started).

Our results suggest that some percentage of ACSC hospitalizations are likely discretionary and responsive to patient behavior and local conditions during the COVID-19 pandemic.^26^ Other research has found that ACSC hospitalizations are also associated with patient-level and local, regional, and national-level factors that are associated with the demand for care, and they must be interpreted carefully in these contexts.^12,13^ Therefore, while our results suggest that there were likely not large decreases in the quality of ambulatory care provided during the pandemic, they also highlight the limitations of using ACSC hospitalization rates as a proxy for the quality of ambulatory care when there are simultaneous changes occurring that may affect the demand for and supply of medical care.

### Limitations

This study has several limitations. Our data included a commercially insured population of adults who were not necessarily representative of the entire US population. The study cohort’s overall rate of ACSC hospitalizations, 4.4 per 1000 enrollees or 440 per 100 000 in the prepandemic period, was lower than the national mean rate,^27,28^ which suggests that the population in our study was younger and healthier. Our results therefore may not be generalizable to the entire US population. The percentage of total hospitalizations classified as ACSCs in our data was similar to that reported in prior literature for the commercially insured.^27^ Our results also do not include hospitalizations among adults who lost or changed their insurance during the pandemic; however, total enrollment decreased only by 2.7% between the prepandemic and pandemic periods, suggesting that there were unlikely to be major shifts in the composition of the enrolled population during the study period. Finally, our data do not include important social and demographic covariates, such as race and ethnicity, language spoken, or income, and we are therefore not able to examine whether existing disparities in ACSC hospitalizations across these characteristics changed during the pandemic.

## Conclusions

We found that rates of ACSC hospitalizations decreased during the pandemic relative to the prepandemic period, with no evidence that patients hospitalized for ACSCs during the pandemic required more intensive medical care than during the prepandemic period. The causes of the decreases in ACSC hospitalizations are likely multifactorial, and we would caution researchers and policy makers from interpreting the changes in ACSC rates seen during the pandemic as solely due to changes in the quality of ambulatory care. Future research should seek to identify the measures of the quality of ambulatory care that are more robust to changes in other patient- and system-level factors that are associated with patient outcomes.

## Notes

Supplement. **eFigure.** Analytic Cohort Construction **eTable 1.** Algorithms for Identifying Inpatient Hospitalizations **eTable 2.** Adjusted Risk Ratios of ACSC Hospitalizations Prepandemic Relative to Postpandemic for Individuals Under 65 **eTable 3.** Characteristics of ACSC Hospitalizations Prepandemic Relative to Postpandemic for Individuals Under 65 **eTable 4.** Adjusted Risk Ratios of ACSC Hospitalizations Prepandemic Relative to Postpandemic Excluding March to May **eTable 5.** Characteristics of ACSC Hospitalizations Prepandemic Relative to Postpandemic Excluding March to May **eTable 6.** Adjusted Odds Ratios of ACSC Hospitalizations Pre-pandemic Relative to Post-pandemic for Individuals Continuously Enrolled From March 2019 - February 2021 **eTable 7.** Characteristics of ACSC Hospitalizations Prepandemic Relative to Postpandemic for Individuals Continuously Enrolled Between March 2019 - February 2021
