# Supplementary material for: Trends in Hospitalizations for Ambulatory Care–Sensitive Conditions During the COVID-19 Pandemic
Source: JAMA Netw Open. 2022 Mar 17;5(3):e222933. doi: 10.1001/jamanetworkopen.2022.2933 (PMC8931555; doi:10.1001/jamanetworkopen.2022.2933)
Supplement: Supplement. — eFigure. Analytic Cohort Construction eTable 1. Algorithms for Identifying Inpatient Hospitalizations eTable 2. Adjusted Risk Ratios of ACSC Hospitalizations Prepandemic Relative to Postpandemic for Individuals Under 65 eTable 3. Characteristics of ACSC Hospitalizations Prepandemic Relative to Postpandemic for Individuals Under 65 eTable 4. Adjusted Risk Ratios of ACSC Hospitalizations Prepandemic Relative to Postpandemic Excluding March to May eTable 5. Characteristics of ACSC Hospitalizations Prepandemic Relative to Postpandemic Excluding March to May eTable 6. Adjusted Odds Ratios of ACSC Hospitalizations Pre-pandemic Relative to Post-pandemic for Individuals Continuously Enrolled From March 2019 - February 2021 eTable 7. Characteristics of ACSC Hospitalizations Prepandemic Relative to Postpandemic for Individuals Continuously Enrolled Between March 2019 - February 2021 [file jamanetwopen-e222933-s001.pdf]

Supplementary Online Content

Becker NV, Karmakar M, Tipirneni R, Ayanian JZ. Trends in hospitalizations for ambulatory care–sensitive conditions during the COVID-19 pandemic. *JAMA Netw Open.* 2022;5(3):e222933. doi:10.1001/jamanetworkopen.2022.2933

eFigure. Analytic Cohort Construction
eTable 1. Algorithms for Identifying Inpatient Hospitalizations
eTable 2. Adjusted Risk Ratios of ACSC Hospitalizations Prepandemic Relative to Postpandemic for Individuals Under 65
eTable 3. Characteristics of ACSC Hospitalizations Prepandemic Relative to Postpandemic for Individuals Under 65
eTable 4. Adjusted Risk Ratios of ACSC Hospitalizations Prepandemic Relative to Postpandemic Excluding March to May
eTable 5. Characteristics of ACSC Hospitalizations Prepandemic Relative to Postpandemic Excluding March to May
eTable 6. Adjusted Odds Ratios of ACSC Hospitalizations Pre-pandemic Relative to Post-pandemic for Individuals Continuously Enrolled From March 2019 - February 2021
eTable 7. Characteristics of ACSC Hospitalizations Prepandemic Relative to Postpandemic for Individuals Continuously Enrolled Between March 2019 - February 2021

This supplementary material has been provided by the authors to give readers additional information about their work.

© 2022 Becker NV et al. *JAMA Network Open.*

eFigure. Analytic Cohort Construction

Figure A1: Analytic cohort construction

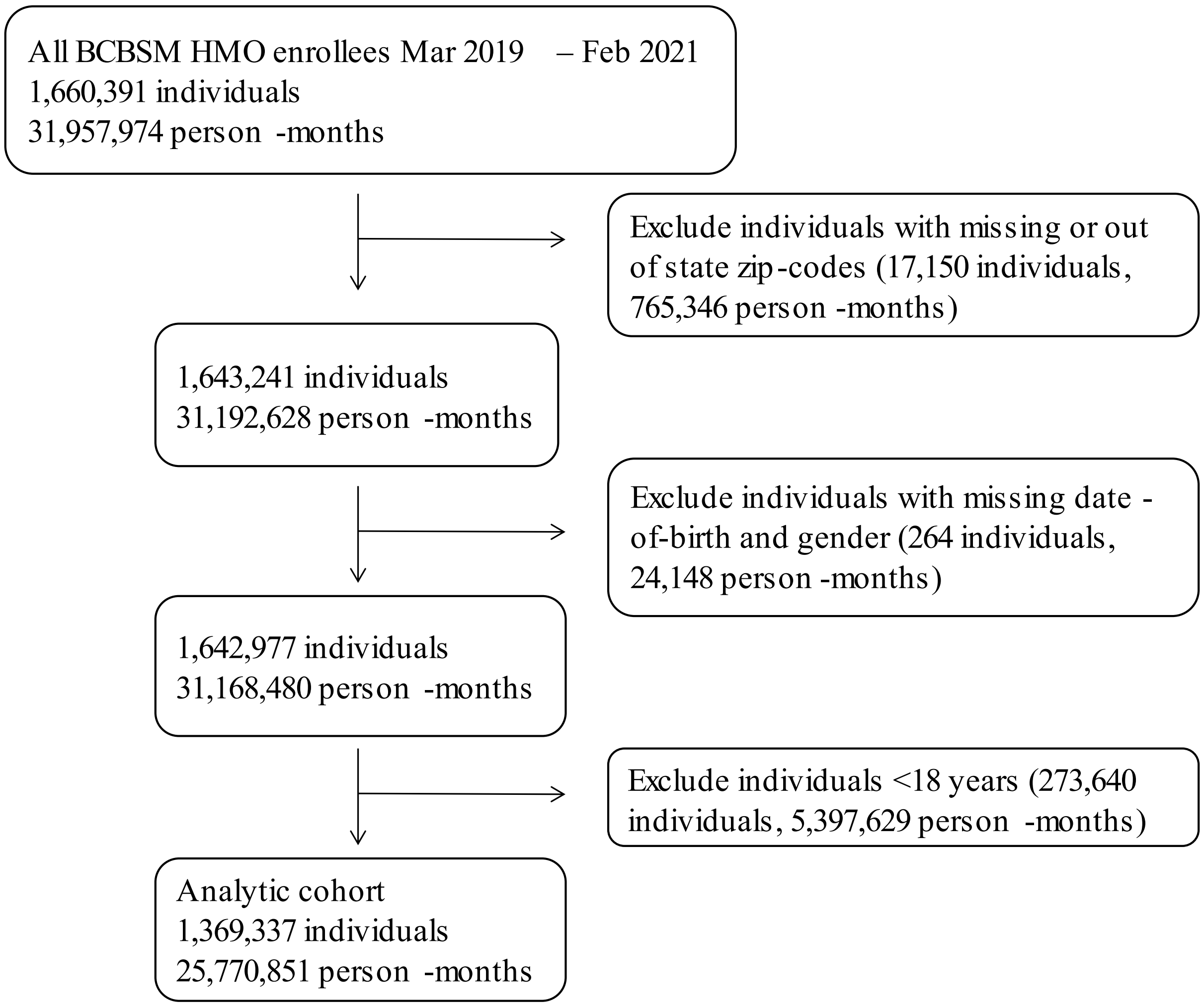

© 2022 Becker NV et al. *JAMA Network Open.*

eTable 1. Algorithms for Identifying Inpatient Hospitalizations

Type of hospitalization	Algorithm	Source
Any inpatient hospitalization	(1) Bill Type = 11 or 12 and (2) DRG is not a rehab code (945, 946, 949, 950) and (3) Revenue code is not an IP rehab code (118, 128, 138, 148, 158)	Ellimootil et. al. 2017
COVID-19 hospitalization	ICD-10 code U071 present on an inpatient claim in any position (admitting diagnosis, principal diagnosis, or any one of 25 additional variables for additional ICD-10 diagnostic codes).	n/a
ICU hospitalization	Revenue codes 173, 174, 200, 201, 202, 203, 204, 206, 207, 208, 209, 210, 211, 212, 213, 214, 219, 233, or 234	Weissman et. al. 2017
ACSC hospitalizations	AHRQ ACSC inclusion/exclusion criteria were used, with the following adaptations for commercial claims data:<br>1) The BCBS claims data included both an "Admission Diagnosis" and "Principal Diagnosis" field which could differ, while the AHRQ algorithm only includes a "Principal Diagnosis" code, so hospitalizations were assigned as ACSC hospitalizations if either the admitting or principal diagnosis codes were one the qualifying ICD-10 codes.<br>2) The ACSC algorithm includes some exclusions based on admission source and point of origin variables in Medicare claims. These were mapped to admission source codes in the BCBS claims, which do not include a point of origin variable.	AHRQ ACSC algorithms, available at https://www.qualityindicators.ahrq.gov/Archive/PQI_TechSpec_ICD10_v2019.aspx

© 2022 Becker NV et al. *JAMA Network Open.*

**eTable 2.** Adjusted Risk Ratios of ACSC Hospitalizations Prepandemic Relative to Postpandemic for Individuals Under 65					
	Any non-COVID non-ACSC hospitalization	Any ACSC hospitalization	Respiratory ACSC hospitalization	Diabetes ACSC hospitalization	Other ACSC hospitalization
Unit of analysis	Person-months	Person-months	Person-months	Person-months	Person-months
N	22,041,378	22,004,318	21,646,843	21,783,236	21,643,344
Adjusted risk ratio in post-pandemic relative to pre-pandemic period (95% CI)	0.85 (0.83 - 0.86)	0.74 (0.69 - 0.79)	0.49 (0.44 - 0.56)	0.87 (0.77 - 0.98)	0.89 (0.79 - 0.99)
P-value	p<0.001	p<0.001	p<0.001	p = 0.02	p = 0.03
Each model uses data at the person-month level restricted to age < 65, and displays the adjusted risk ratio that an individual enrolled in a given month has a hospitalization in one of the following categories (non-ACSC non-COVID, ACSC, respiratory ACSC, diabetes ACSC, or other ACSC) in the pre-pandemic period (March 2019 - February 2020) compared with the post-pandemic period (March 2020 - February 2021). Additional covariates include age, gender, and a set of calendar month and Michigan county of residence fixed effects.					

© 2022 Becker NV et al. *JAMA Network Open.*

**eTable 3.** Characteristics of ACSC Hospitalizations Prepandemic Relative to Postpandemic for Individuals Under 65			
	ICU stay for ACSC hospitalizations		LOS for ACSC hospitalization
Unit of analysis	ACSC admission		ACSC admission
N	3,948		3,948
Adjusted risk ratio in post-pandemic relative to pre-pandemic period (95% CI)	1.05 (0.97 - 1.13)	Adjusted incidence rate ratio in post-pandemic relative to pre-pandemic period (95% CI)	1.02 (0.95 - 1.09)
P-value	p = 0.22		p = 0.61
Each model uses data at the ACSC admission level for ACSC admissions among individuals < age 65 and displays the adjusted risk ratio than an ACSC hospitalization includes an ICU stay (Column 2) and the adjusted incidence rate ratio of the length of stay of that ACSC admission (Column 4) in the pre-pandemic period (March 2019 - February 2020) compared with the post-pandemic period (March 2020 - February 2021). Additional covariates include age, gender, and a set of calendar month of admission fixed effects.			

© 2022 Becker NV et al. *JAMA Network Open.*

**eTable 4.** Adjusted Risk Ratios of ACSC Hospitalizations Prepandemic Relative to Postpandemic Excluding March to May					
	Any non-COVID non ACSC hospitalization	Any ACSC hospitalization	Respiratory ACSC hospitalization	Diabetes ACSC hospitalization	Other ACSC hospitalization
Unit of analysis	Person-months	Person-months	Person-months	Person-months	Person-months
N	19,333,772	19,325,176	19,127,098	19,060,145	19,251,197
Adjusted risk ratio in post-pandemic relative to pre-pandemic period (95% CI)	0.86 (0.85 - 0.87)	0.73 (0.69 - 0.77)	0.49 (0.45 - 0.54)	0.96 (0.86 - 1.07)	0.89 (0.79 - 0.99)
P-value	p<0.001	p<0.001	p<0.001	p = 0.460	p = 0.03
Each model uses data at the person-month level and displays the adjusted risk ratio that an individual enrolled in a given month has a hospitalization in one of the following categories (non-ACSC non-COVID, ACSC, respiratory ACSC, diabetes ACSC, or other ACSC) in the pre-pandemic period (May 2019 - February 2020) compared with the post-pandemic period (May 2020 - February 2021), excluding the calendar months of March, April, and May in both years. Additional covariates include age, gender, and a set of calendar month and Michigan county of residence fixed effects.					

© 2022 Becker NV et al. *JAMA Network Open.*

eTable 5. Characteristics of ACSC Hospitalizations Prepandemic Relative to Postpandemic Excluding March to May

	ICU stay for ACSC hospitalizations		LOS for ACSC hospitalization
Unit of analysis	ACSC admission		ACSC admission
N	7,051		7,051
Adjusted risk ratio in post-pandemic relative to pre-pandemic period (95% CI)	0.99 (0.93 - 1.05)	Adjusted incidence rate ratio in post-pandemic relative to pre-pandemic period (95% CI)	1.04 (1.00 - 1.10)
P-value	p = 0.729		p = 0.072

Each model uses data at the ACSC admission level for ACSC admissions, and displays the adjusted risk ratio than an ACSC hospitalization includes an ICU stay (Column 2) and the adjusted incidence rate ratio of the length of stay of that ACSC admission (Column 4) in the pre-pandemic period (May 2019 - February 2020) compared with the post-pandemic period (May 2020 - February 2021), excluding the calendar months of March, April, and May in both years. Additional covariates include age, gender, and a set of calendar month of admission fixed effects.

© 2022 Becker NV et al. *JAMA Network Open.*

**eTable 6.** Adjusted Odds Ratios of ACSC Hospitalizations Pre-pandemic Relative to Post-pandemic for Individuals Continuously Enrolled From March 2019 - February 2021					
	Any non-COVID non ACSC hospitalization	Any ACSC hospitalization	Respiratory ACSC hospitalization	Diabetes ACSC hospitalization	Other ACSC hospitalization
Unit of analysis	Person-months	Person-months	Person-months	Person-months	Person-months
N	17,753,400	17,721,998	17,648,776	17,564,739	17,667,677
Adjusted odds ratio in post-pandemic relative to pre-pandemic period (95% CI)	0.68 (0.67 - 0.69)	0.62 (0.59 - 0.64)	0.47 (0.43 - 0.51)	0.76 (0.69 - 0.85)	0.68 (0.63 - 0.72)
P-value	p<0.001	p<0.001	p<0.001	p<0.001	p<0.001

Each model uses data at the person-month level, restricted to individuals continuously-enrolled in a BCBSM HMO plan between March 2019 - February 2021, and displays the odds ratio that an individual enrolled in a given month has a hospitalization in one of the following categories (non-ACSC non-COVID, ACSC, respiratory ACSC, diabetes ACSC, or other ACSC) in the pre-pandemic period (March 2019 - February 2020) compared with the post-pandemic period (March 2020 - February 2021). Additional covariates include age, gender, and a set of calendar month and Michigan county of residence fixed effects. Note that unlike the other results reported in this manuscript, these analyses were run as logistic regressions and thus report odds ratios, not risk ratios. This was done due to difficulties with convergence running the analyses as generalized linear regression models.

© 2022 Becker NV et al. *JAMA Network Open.*

**eTable 7.** Characteristics of ACSC Hospitalizations Prepandemic Relative to Postpandemic for Individuals Continuously Enrolled Between March 2019 - February 2021			
	ICU stay for ACSC hospitalizations		LOS for ACSC hospitalization
Unit of analysis	ACSC admission		ACSC admission
N	8,763		8,763
Adjusted odds ratio in post-pandemic relative to pre-pandemic period (95% CI), p-value	0.98 (0.90 - 1.08), p = 0.716	Adjusted incidence rate ratio in post-pandemic relative to pre-pandemic period (95% CI), p-value	1.03 (0.99 - 1.08), p = 0.134
P-value			
Each model uses data at the ACSC admission level for ACSC admissions among individuals continuously enrolled in a BCBSM HMO plan between March 2019 - February 2021, and displays the adjusted risk ratio than an ACSC hospitalization includes an ICU stay (Column 2) and the adjusted incidence rate ratio of the length of stay of that ACSC admission (Column 4) in the pre-pandemic period (March 2019 - February 2020) compared with the post-pandemic period (March 2020 - February 2021). Additional covariates include age, gender, and a set of calendar month of admission fixed effects. Note that unlike the other results reported in this manuscript, the results reported in Column 2 were run as logistic regressions and thus report a odds ratio, not a risk ratio. This was done due to difficulties with convergence running the analysis as a generalized linear regression model.			

© 2022 Becker NV et al. *JAMA Network Open.*
